# Measurements of ultrafast spin-profiles and spin-diffusion properties in the domain wall area at a metal/ferromagnetic film interface

**DOI:** 10.1038/s41598-017-15234-7

**Published:** 2017-11-08

**Authors:** T. Sant, D. Ksenzov, F. Capotondi, E. Pedersoli, M. Manfredda, M. Kiskinova, H. Zabel, M. Kläui, J. Lüning, U. Pietsch, C. Gutt

**Affiliations:** 1 Department Physik, Universität Siegen, Walter-Flex-Strasse 3, D-57072 Siegen, Germany; 2 FERMI, Elettra-Sincrotrone Trieste, 34149 Basovizza, Trieste Italy; 3 Institut für Physik, Johannes Gutenberg Universität Mainz, D-55099 Mainz, Germany; 4 Sorbonne Universités, UPMC Univ Paris 06, CNRS, Laboratoire de Chimie Physique - Matière et Rayonnement, 75005 Paris, France

## Abstract

Exciting a ferromagnetic material with an ultrashort IR laser pulse is known to induce spin dynamics by heating the spin system and by ultrafast spin diffusion processes. Here, we report on measurements of spin-profiles and spin diffusion properties in the vicinity of domain walls in the interface region between a metallic Al layer and a ferromagnetic Co/Pd thin film upon IR excitation. We followed the ultrafast temporal evolution by means of an ultrafast resonant magnetic scattering experiment in surface scattering geometry, which enables us to exploit the evolution of the domain network within a 1/e distance of 3 nm to 5 nm from the Al/FM film interface. We observe a magnetization-reversal close to the domain wall boundaries that becomes more pronounced closer to the Al/FM film interface. This magnetization-reversal is driven by the different transport properties of majority and minority carriers through a magnetically disordered domain network. Its finite lateral extension has allowed us to measure the ultrafast spin-diffusion coefficients and ultrafast spin velocities for majority and minority carriers upon IR excitation.

## Introduction

Nanoscale magnetization reversal is a key component of modern information technology with the speed limits of technologies such as magnetic fields or driven spin-currents limited to the 100 ps time scale^1,2^. The search for faster switching mechanisms has been one of the science drivers behind ultrafast demagnetization with femtosecond laser excitation^3^–^12^. As recently demonstrated in Ref.^13^ one can broadly categorize the field of ultrafast demagnetization into two areas: demagnetization by hot electrons and all-optical demagnetization including also switching. Whereby the underlying physics of ultrafast demagnetization is still discussed with spin-flips induced by spin-orbit interaction^14^, Elliot-Yafet scattering^15^ or transport by means of superdiffusive processes^2^ are being recognized as important mechanisms. It adds to the complexity of the field, that the relative contribution of the processes involved are highly material dependent^13^. Such a material dependence implies that ultrafast spin-diffusion, for example across interfaces or in chemical inhomogeneous materials, yields transient states of non-homogeneous magnetization. Calculations of ultrafast spin flow in layered heterostructures gave first indications that at metal/FM interfaces spin-profiles can be spatially structured due to differences in spin transport properties^16^. Recently, ultrafast resonant X-ray diffraction from GdFeCo intermetallics revealed indeed a nanoscale spin reversal^17^ caused by different transport properties due to chemical and magnetic nanoscale inhomogeneities. In ferromagnets an ultrafast lateral nanoscale transport of angular momentum has been observed in a perpendicular Co/Pd thin film exhibiting  a maze like domain structure^7^, highlighting the influence of lateral magnetic inhomogeneities on spin-transport properties. The examples show that measurements of ultrafast spin transport properties are needed for a deeper understanding of the complexity of ultrafast demagnetization phenomena and the physics involved, especially in non-homogeneous materials.

Here we report surface sensitive XUV-laser diffraction experiments that probe the optically excited domain structure of a Co/Pd thin film close to an interface with a metallic layer. The necessary control of the penetration depths of the XUV-rays was attained via varying the incident angles and comparing the corresponding resonant magnetic diffraction patterns. We observe a transient nanoscale magnetization-reversal close to the domain wall boundaries which decays on ps time scales. This microscopic in nature magnetization-reversal is directly linked to the different transport properties of laser excited majority and minority carriers across the magnetic domain boundaries. The lateral extension of the magnetization-reversal area turns out to be highly sensitive to the magnitude of the spin diffusion constants which has allowed us to determine with high precision their temporal evolution after laser excitation. We find that the diffusion properties of majority and minority carriers depend on the distance from the FM/metal interface. Accordingly, the degree and extension of the observed magnetization-reversal area appear depth dependent with magnetization-reversal becoming more pronounced closer to the FM/metal interface.

## Results

**Figure 1:**
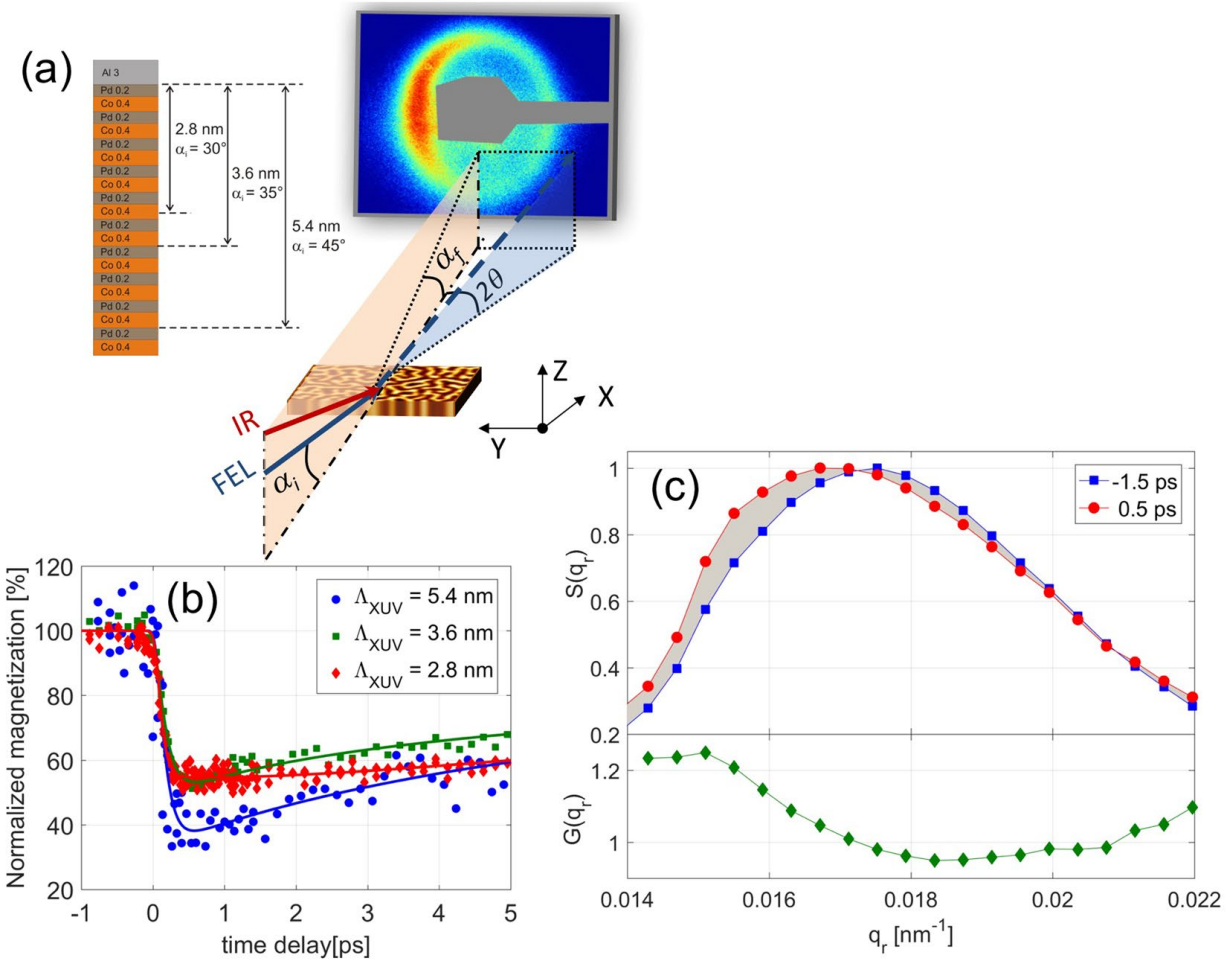
Experimental pump-probe setup in reflection geometry, demagnetization and structure factor. (**a**) XUV pulses incident at fixed angles *α*_i_ probe the magnetic multilayer sample in penetration depths ranging from 2.8 to 5.4 nm. The IR pump pulse arrives with a small offset (<2°) w.r.t. the FEL beam on the sample. The CCD measures the resonant magnetic scattering signal where the intense specularly reflected beam is blocked by the beamstop and the IR radiation is absorbed by an IR filter (not shown) in front of the CCD. *α*_f_ and 2*θ* are the out-of-plane and in-plane scattering angles, respectively. (**b**) The time evolution of the normalized magnetization (measured as the area under the scattering peak). The lines are fits to the data points following Ref.^7^. (**c**) Upper panel: The scattering structure factor $S({q}_{r})$ (normalized to its maximum) for an unpumped (blue, $\tau$ = −1.5 ps) and for a pumped film (red, $\tau$ = 0.5 ps) at ${{\rm{\Lambda }}}_{XUV}$ = 3.6 nm. The lower panel shows the corresponding distortion function $G({q}_{r})=\frac{S({q}_{r})}{{S}_{0}({q}_{r})}$.

The experimental set-up for studying the spatio-temporal response of a lateral labyrinth domain pattern in a thin Co/Pd film is shown schematically in Fig. 1. In Co/Pd films alternating ‘up’ and ‘down’ magnetic domains form in a self-organized manner with the magnetization direction parallel or antiparallel to the surface normal. The sample is chemically homogeneous in the lateral direction and the boundaries between the domains are of pure magnetic origin. Circularly polarized XUV rays^18^ are tuned to the Co M_2,3_ dichroic transition, which exhibits magnetic scattering contrast due to the X-ray magnetic circular dichroism effect^19–21^. The XUV-rays hit the sample at angles of 45, 35, and 30 degrees with corresponding XUV penetration depths of Λ_XUV_ = 5.4, 3.6 and 2.8 nm inside the magnetic multilayer structure (without the Al cap layer), respectively. Due to the disordered domain structure we observe the typical ring-like diffraction feature well known from transmission SAXS experiments representing a broad distribution of Fourier components of the magnetic structure^7^. A small angular tilt of the magnetization direction with respect to the plane defined by the incoming beam or a chiral component in the domain wall magnetization^22^ leads to the apparent asymmetry of the pattern. The sample is pumped with a 100 fs IR laser pulse of 780 nm wavelength impinging on the sample with a small 2 degree offset with respect to the XUV beam.

We evaluate the radial scattering intensity distribution after azimuthal integration and normalization to the $1/{q}_{z}^{4}$ Fresnel reflectivity^23^ where ${q}_{z}$ denotes the wavevector transfer perpendicular to the sample surface. The scattered intensity is presented as a function of ${q}_{r}=\,\sqrt{\,{q}_{x}^{2}+{q}_{y}^{2}}\,$, where ${q}_{x}$ and ${q}_{y}$ denote the wavevector transfers of directions within the sample plane. The area of the scattering peak is a measure for the average domain magnetization of the ferromagnetic film.

In Fig. 1(b) we plot the normalized magnetization magnitude as a function of pump-probe delay. All three measurements were performed with the same incident IR fluence of 11 mJ/cm^2^. Taking the angular dependence of the IR penetration depths and IR reflectivity into account we calculate the IR power deposited within the three XUV penetration depths to be 6.4% (Λ = 2.8 nm), 7.7% (Λ = 3.6 nm) and 10.6% (Λ = 5.4 nm)of the incident IR power, respectively (see also Supplementary Figure S3 and Table ST1). Thus, the stronger demagnetization observed for Λ_XUV_ = 5.4 nm can be attributed to the larger amount of deposited IR energy. The time constants of demagnetization are (121 ± 20) fs, (140 ± 20) fs and (146 ± 25) fs for the penetration depths of 2.8, 3.6 and 5.4 nm, respectively (Fig. S3). While the magnetization amplitude is proportional to the area under the scattering peak, its spatial arrangement is encoded in the line shape of the in-plane structure factor $S({q}_{r})$. An equilibrium ${S}_{0}({q}_{r})$ and the corresponding IR excited $S({q}_{r})$ at a delay time of 500 fs are shown in Fig. 1(c) for a penetration depth of 3.6 nm with both curves normalized to their respective maximum. Transmission SAXS experiments^24^ and holographic X-ray imaging^25^ showed that spin diffusion is the main contribution to ultrafast changes in magnetic domain patterns. Here, the ultrafast spin diffusion into the domain interface regions reduces the high-q $S({q}_{r})$ contributions with an apparent shift of the maximum of $S({q}_{r})$ towards smaller ${q}_{r}$-values. To visualize and analyze the spatial details of this diffusion process we introduce a distortion function $G({q}_{r})$ which is the ratio of excited and equilibrium structure factor $G({q}_{r})\,=\,\frac{S({q}_{r})}{{S}_{0}\,({q}_{r})}$. This function is in close analogy to a Debye-Waller factor in crystallography measuring the Fourier amplitude of the electron density smearing due to thermal vibrations - in our case $G({q}_{r})$ measures the Fourier components of the spatial evolution of the spin-profile in the domain boundary area. Diffusion of majority carriers into the domain area of opposite magnetization direction leads to a broadening of the domain walls associated with a monotonic Gaussian decay of the distortion function $G({q}_{r})\,\propto \,exp(-M\,{q}_{r}^{2})$. However, the observed $G({q}_{r})$ clearly deviates from this monotonic behavior displaying a shallow minimum at ${q}_{r}=0.018$ nm^−1^ and an increase of $G({q}_{r})$ at larger ${q}_{r}$-values. This behavior indicates the presence of an additional real space correlation length in the excited spin-system.

**Figure 2:**
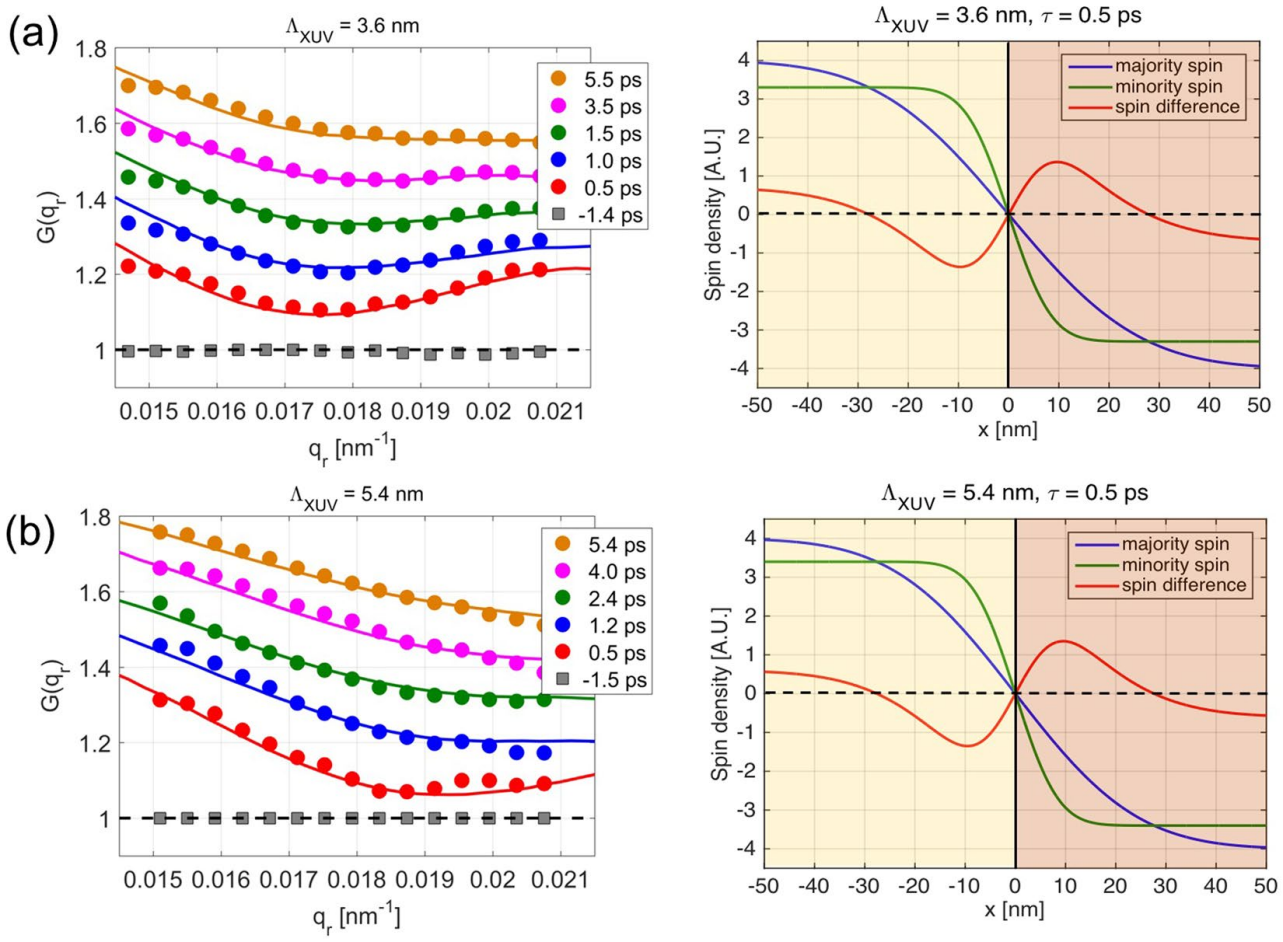
Measured and calculated distortion functions and spin difference profiles. The left panels display the distortion functions from the measured azimuthally structure factors (filled symbols) and the corresponding calculated distortion functions (solid lines) following the model described in the SI for the XUV penetration depths. The distortion functions for different delays for each penetration depth are vertically shifted for clarity. The right panels show the majority spin profile (${m}_{\uparrow }(x)$), minority spin profile (${m}_{\downarrow }(x)$) and the spin difference profile (${m}_{\uparrow }(x)\,-{m}_{\downarrow }(x)$) for a pump-probe delay of $\tau$ = 0.5 ps. The spin up domain (Co↑) exists for $x\, <$ 0 and the spin down domain (Co↓) for $x\, >$ 0 with the domain boundary at $x\,$= 0.

The temporal response of $G({q}_{r})$ following the laser excitation is shown in Fig. 2 for two different penetration depths. The panels (a) and (b) reveal that the shape of $G({q}_{r})$ depends on the value of the time delay, the depth probed and the IR power deposited inside the magnetic material. Smaller penetration depths and shorter time delays exhibit a more pronounced modulation of $G({q}_{r})$ (see also Supplementary Material), while for larger penetration depths and time scales approaching the 4–5 ps range $G({q}_{r})$ resembles the monotonic decay associated with simple spin diffusion-broadened domain walls.

## Discussion

To understand our findings, we simulated the time-dependent magnetization in a quasi 2-d Al/Co-Pd system based on ultrafast spin-dependent transport in the magnetic multilayer^16,26^. The magnetization configuration  inside the ferromagnetic Co/Pd layer is entirely defined via domains with up/down directions of magnetization. The dynamics of both spin channels is then determined by solving the super-diffusive spin transport equation^2,16^ with the energy dependent life-times of majority and minority spins as input parameters. Upon laser irradiation electrons excited in the Al capping layer are flowing into the FM thin film, and spin polarized electrons from the FM are diffusing into the Al capping layer and within the network of domains. The observed difference in the broadening of the majority and minority spin-profiles across the domain interface is due to the different  spin life times with the majority spin-profile being considerably broader. The difference between the two profiles of both spin-channels exhibit a strong non-monotonic behavior close to the domain wall with an additional length scale in the magnetization profile.

Based on these simulations we calculate the scattering signal from magnetic domains with the broadening of the profiles of the respective spin-species as refinement parameter. For the model, we consider an array of alternating domains in Co with a percentage level variation in their widths to account for the limited long-range order of the disordered maze domain pattern. The spin-profile at the domain walls is modelled by the diffusion broadened function ${m}_{\sigma }(t,x)={\mu }_{\sigma }\cdot {\rm{erf}}(x/({W}_{\sigma }(t)\,+\,{W}_{D}/2\,))$, where $x$ is the lateral sample coordinate, ${\mu }_{\sigma }$ is the magnetic moment and ${W}_{\sigma }(t)$ is the time dependent broadening of the spin-profile which depends on the diffusion properties of the spin carriers ($\sigma$ labels majority or minority carriers). ${W}_{D}$ is the intrinsic width of the domain walls. From the values of anisotropy (K = 1.3 × 10^7^ erg/cm^2^)^27^ and exchange stiffness (A = 1 × 10^−6^ erg/cm) for our films we estimate $DW\approx {\rm{\pi }}\sqrt{A/K}\approx 10\,nm$ and discuss the influence of domain wall thickness on the results in the Supplemental material. From the Fourier transform of the magnetization profile ${m}_{\uparrow }(t,x)-{m}_{\downarrow }(t,x)$ we obtain the in-plane structure factor $S({q}_{r})$ of the IR excited spin-system. The two spin dependent broadening parameters ${W}_{\sigma }(t)$ serve as adjustable parameters while the overall degree of magnetization within a domain is fixed to reproduce the measured value of demagnetization for each penetration depth (see Fig. 1).

The resulting $G({q}_{r})$ are shown as solid lines in the left panels 2(a) and 2(b). The model fits the data quite well with smaller deviations visible at low q-values. A more complex spatial spin-profile than the simple erf(x) is also able to model the low-q data, however given the small number of data points we concentrate here on the simple and instructive erf profile. The majority and minority spin profiles displayed in the right panels of Fig. 2 reflect the different diffusion constants leading to different broadening of the spin density distribution within the domain wall area. The green minority profile is only slightly broadened beyond the intrinsic domain width while the blue majority profile softens over a larger region of width 30 nm. This results in an excess of minority carriers close to the domain wall producing a considerable wide  local magnetization-reversal in the difference magnetization profile. Within a region of 10–20 nm the magnetic moment reverses from a value of +0.9 ${\mu }_{B}$ to a value of −1.3 ${\mu }_{B}$. For a penetration depth of 2.8 nm we find a local value of −1.6 ${\mu }_{B}$. This modelling result is very robust as only magnetization-reversal can reproduce the characteristic change of slope in the $G({q}_{r})$ function.

The mean degree of local magnetization-reversal depends on the depth probed inside the magnetic material. As $G({q}_{r})$ is less modulated for larger penetration depths the amount and extension of magnetization-reversal is also reduced. Close to the Al/FM interface the lateral magnetization-reversal persists up to 5 ps while deeper in the magnetic multilayer it almost vanishes at 5.4 ps. The origin of this local FM magnetization-reversal lies in the different mobility of the spin-species, the more mobile majority carriers flow out of the interface and domain wall region leaving the less mobile minority carriers behind.

The amount and spatial extension of the magnetization-reversal is very sensitive to the profile broadening ${W}_{\sigma }(t)$. Assuming the electronic system to be thermalized at time scales of 500 fs and longer we expect the spin-profile broadening to be governed by standard diffusion dynamics. Then the broadening is given by ${W}_{\sigma }(t)=2\sqrt{{D}_{\sigma }(\tau )\cdot t}\,\,$which enables us to extract the ultrafast spin diffusion coefficients ${D}_{\sigma }(t)$ for both spin species from the measurements. Diffusion coefficients for the majority carriers are (0.35 ± 0.02) nm^2^/fs at 0.5 ps and for the smallest penetration depth of 2.8 nm and decrease to (0.02 ± 0.01) nm^2^/fs at 5.5 ps delay owing to the cooling of the excited electronic system. Comparison to literature is difficult as ultrafast transport properties are notoriously difficult to measure by optical methods without spatial sensitivity. However, we can compare our measured values to estimates from Wieczoreck *et al*.^28^ based on energy averaged values of spin lifetimes and density functional spin-velocities in Co yielding values of 2.4 nm^2^/fs for majority and 0.27 nm^2^/fs for minority carriers.

We detect systematic differences in the diffusion constant for different depths inside the material with the surface region exhibiting slightly faster dynamics than regions deeper inside the material (0.27 nm^2^/fs at 0.5 ps for ${{\rm{\Lambda }}}_{XUV}\,=$ 5.4 nm). For time delays longer than 3 ps the diffusion constants become almost identical within the error bars. Heat and energy flow from the excited Al cap layer to the bulk, mediated by direct absorption of light and by flow of hot electrons from the Al cap layer into the FM multilayer are causing the slightly faster dynamics in the near-surface region at early times^8,29,30^. This difference between near-surface and sub-surface regions vanishes with advancing thermalization and equilibration.

Compared to the majority carriers the dynamics of the minority carriers is considerably slower with diffusion constants of 0.02 nm^2^/fs at 0.5 ps delay which furthers slows down with increasing the delay time. Within the error bars of our experiment we cannot elucidate a similar depth dependence as for the majority carriers. However, the dynamics of the minority carriers is also showing the same slowing down behavior upon thermalization and cooling of the electronic system. We note that the diffusion constants show a very different temporal behavior when compared to the evolution of the overall magnetization (Fig. 1b). While the magnetization is only slightly recovering within the first few ps after excitation, we observe that the diffusion constant is decreasing from 0.35 nm^2^/fs to 0.02 nm^2^/fs in the same time interval. It is important to note that the X-ray data measures the net amount of transported spins into areas of opposite magnetization and thus the fast decrease of the dynamics suggests that the spin-transport vanishes quickly after thermalization of the electronic system with the lattice (1–1.5 ps, Fig. S9) while the overall magnetization is recovering more slowly via cooling of the lattice.

**Figure 3:**
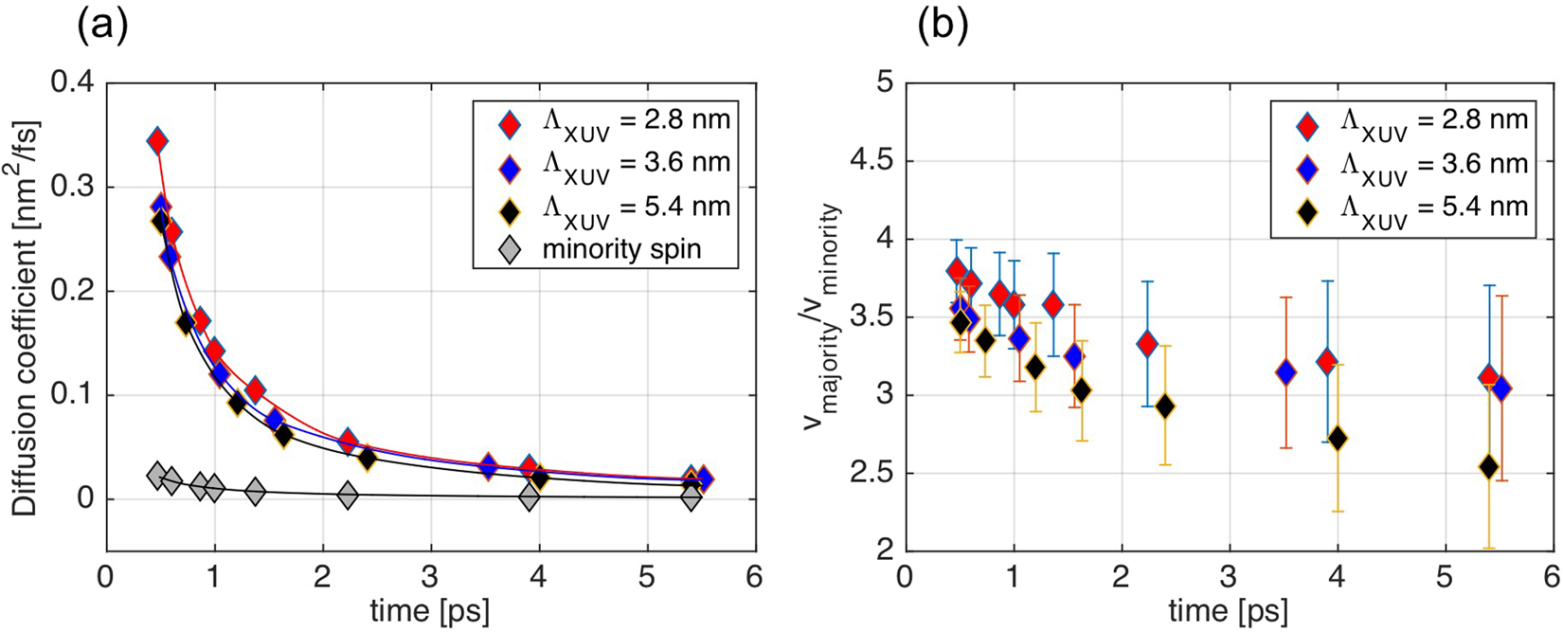
Diffusion coefficients and ratio of velocities for majority and minority spin carriers. (**a**) Diffusion coefficients at selected pump-probe delays for majority and minority spin carriers estimated from the widths of the respective profiles (${m}_{\uparrow }(x)$ and ${m}_{\downarrow }(x)$) across a domain wall. (**b**) Ratio of the velocities of majority and minority spin carriers at selected pump-probe delays. Solid lines are guide to the eyes.

Using the diffusion equation ${D}_{\sigma } \sim {v}_{\sigma }^{2}{\tau }_{\sigma }$ with velocities v and lifetimes ${\rm{\tau }}$ and noticing that the ratio of ${\tau }_{\uparrow }/{\tau }_{\downarrow } \sim 1$ close to the Fermi level in Co^31–33^ we obtain the time dependent ratio of the energy-averaged velocities as $\sqrt{{D}_{\uparrow }/{D}_{\downarrow }} \sim {v}_{\uparrow }/{v}_{\downarrow }.$ This quantity is plotted in Fig. 3b for the different penetration depths. Within the error bar the ratio is quite stable with only a very small time dependence. We find values of ${v}_{\uparrow }/{v}_{\downarrow }$ between 4 and 3.5 which can be compared to the density functional calculations of Wieczorek^28^ predicitng a ratio of 3.

By measuring femtosecond scattering dynamics in a surface sensitive reflection geometry we demonstrate nanoscale and ultrafast lateral magnetization-reversal in the interface region of a metallic Al layer and a ferromagnetic thin film. We find that local nanoscale magnetization-reversal occurs on ultra-fast timescales driven by the different transport properties of majority and minority carriers across FM domain walls. The magnetic properties of the magnetization-reversal such as the locally reversed magnetic moment, its lateral extension and its temporal behavior are very sensitive to the spin transport properties of the material. We find that the transport properties vary with distance from the metal/FM interface highlighting the importance of understanding the flow of energy and spins across interfaces in multilayer structures. Our results suggest that the magnetization-reversal in FM thin films can be tailored to a certain extent. Moreover, the magnetization-reversal is a sensitive experimental indicator for transport properties in IR excited thin films which are otherwise difficult or impossible to measure. This also allows one to experimentally test a variety of theoretical calculations in the context of ultrafast magnetization dynamics.

## Methods

### Sample fabrication

The magnetic multilayer samples studied here have been grown by DC magnetron sputtering on a Si wafer covered by a 50 nm thick Si_3_N_4_ film. The (Co 0.4 nm/Pd 0.2 nm) × 30 multilayer film is grown on a 3 nm Al buffer on the Si_3_N_4_ membrane. A thin Al layer of 3 nm thickness on top of the multilayer is deposited to prevent oxidation.

### Pump-probe measurements

The measurements have been performed at the DiProI beamline^34,35^ at the FERMI FEL facility in Trieste, Italy^36,37^. For the experiment the FEL was tuned to the Co M_2,3_ dichroic transition at 20.8 nm wavelength, pulse duration 50–60 fs, repetition rate 10 Hz and maximum pulse energy of 20 μJ. Using a Kirkpatrick-Baez (KB) optics the beam was focused down to a size of 230(H) × 275(V) μm^2^. Attenuators allow to adjust the photon flux so that the experiments can be performed below the damage threshold of the multilayer sample. The optical laser for pump-probe experiments is the same as the Ti:sapphire seed laser used for generating the FEL pulses in the HGHG scheme and therefore it is intrinsically synchronized to the XUV-FEL pulses with a jitter of less than 10 fs. We used as a pump a 780 nm IR pulse of 100 fs duration and size 380(H) × 270(V) μm^2^. Time delays of ± 570 ps can be achieved by a translation stage. The maximum delay in this experiment was 5 ps.

Ultrafast resonant magnetic scattering in reflection mode was employed as a probe technique. Three pump-probe experiments with incident angles of 30, 35 and 45 degrees have been performed with corresponding XUV penetration depths of ${{\rm{\Lambda }}}_{XUV}\,=$ 2.8 nm, 3.6 nm and 5.4 nm inside the magnetic multilayer, respectively. The scattered radiation is detected using a charge coupled device (CCD) area detector (2048 × 2048 pixel, 13.5 × 13.5 μm^2^ pixel size) located at a distance of 10.2 cm from the sample. The intense reflected primary beam is blocked by a beam stop. The CCD is IR protected by an Al filter.

### Data treatment

For each time delay 200 scattering patterns with right circular polarized radiation have been measured. The patterns have been normalized to the incoming flux, averaged and binned by a factor of 2. Areas around the beamstop and charge scattering streaks have been masked. Additional charge signal has been removed by determining for each time delay a separate background signal based on fitting and extraploating the low q signal. Each pixel on the camera is then attributed to a triple of (${q}_{x},{q}_{y},{q}_{z}$) values of the corresponding wavevector transfers. The scattering signal is multiplied with ${q}_{z}^{4}$ to account for the Fresnel reflectivity which allows to extract the reflection SAXS signal $S({q}_{r})$ as a function of radial wavevector transfer ${q}_{r}=\,\sqrt{\,{q}_{x}^{2}+{q}_{y}^{2}}\,\,$which lies in the plane of the magnetic domain network. The distortion function $G({q}_{r})\,=\,\frac{S({q}_{r})}{{S}_{0}\,({q}_{r})}$ is evaluated within the main intensity profile of the magnetic scattering peak thus minimizing parasitic contributions.

The average magnetization of a domain is extracted from the numerical integration of the structure peak $S({q}_{r})\,\,$peak. The resulting curves of time dependent magnetization are fitted by the equation^38^

$$\frac{{\rm{\Delta }}M}{M}=[(\frac{{A}_{1}}{{(t/{\tau }_{0}+1)}^{1/2}}\,-\,\frac{({A}_{2}{\tau }_{E}\,-\,{A}_{1}{\tau }_{M}){e}^{-\frac{t}{{\tau }_{M}}}}{{\tau }_{E}\,-\,{\tau }_{M}}\,-\,\frac{{\tau }_{E}({A}_{1}\,-\,{A}_{2}){e}^{-\frac{t}{{\tau }_{M}}}}{{\tau }_{E}\,-\,{\tau }_{M}}){\rm{\Theta }}(t)]\otimes {\rm{\Gamma }}(t)$$

where *M* is the unpumped magnetization, ${\rm{\Delta }}M$ is the change in the magnetization. ${A}_{1}$ and ${A}_{2}$ are constants. ${\tau }_{M}\,$is the demagnetization time and ${\tau }_{E}$ is the time constant for the partial recovery of the magnetization. ${\rm{\Theta }}(t)$ is a step function and ${\rm{\Gamma }}(t)$ is the Gaussian laser pulse. ${\tau }_{E}$ values have been found to be $\,{\tau }_{E}\,=$ (10 ps, 10 ps, 20 ps) for the three different incident angles of (30, 35, 45).

### Simulation of scattering patterns

The unpumped scattering pattern is modelled by 5000 magnetic domains with varying positive and negative magnetization. The limited long range order of the labyrinth domain network is realized by allowing for random variations (size variation 10%) of the domain size around a main domain size of 190 nm. Fourier transformation of the resulting real space spin-profile yields the magnetic scattering amplitude of the unpumped system. Based on this realization of a magnetic domain arrangement the spin profiles at the domain wall boundaries are modified using error function profiles ${m}_{\sigma }(t,x)={\mu }_{\sigma }\cdot {\rm{erf}}(x/({W}_{\sigma }(t)\,+\,DW/2\,))\,$for majority ${m}_{\uparrow }(x)$ and minority spin profiles ${m}_{\uparrow }(x).\,\,$In order to account for the limited spatial correlation length in the pumped case the widths ${W}_{\sigma }(t)$ of the respective profiles are also subject to small random fluctuations (10%) around their mean values. An ensemble averaged distortion profile is finally produced by calculating and averaging 500 scattering patterns for each time delay.

### Data availability

The datasets generated during and/or analysed during the current study are available from the corresponding author on reasonable request.

## Electronic supplementary material

Supplementary Information
