# Supplementary material for: Measurements of ultrafast spin-profiles and spin-diffusion properties in the domain wall area at a metal/ferromagnetic film interface
Source: Sci Rep. 2017 Nov 8;7:15064. doi: 10.1038/s41598-017-15234-7 (PMC5678147; doi:10.1038/s41598-017-15234-7)
Supplement: Supplementary file 1 — Supplementary Information [file 41598_2017_15234_MOESM1_ESM.pdf]

Measurements of ultrafast spin-profiles and spin-diffusion properties in the domain wall area at a metal/ferromagnetic film interface

T. Sant[1], D. Ksenzov[1], F. Capotondi[2], E. Pedersoli[2], M. Manfredda[2], M. Kiskinova[2], H. Zabel[3], M. Kläui[3], J. Lüning[4], U. Pietsch[1] and C. Gutt[1*]

[1]Department Physik, Universität Siegen, Walter-Flex-Strasse 3, D-57072 Siegen, Germany
[2]FERMI, Elettra-Sincrotrone Trieste, 34149 Basovizza, Trieste, Italy
[3]Institut für Physik, Johannes Gutenberg Universität Mainz, D-55099 Mainz, Germany
[4]Sorbonne Universités, UPMC Univ Paris 06, CNRS, Laboratoire de Chimie Physique - Matière et Rayonnement, 75005 Paris, France

** email: gutt@physik.uni-siegen.de*

Supplementary information

1. Resonant magnetic scattering in reflection geometry

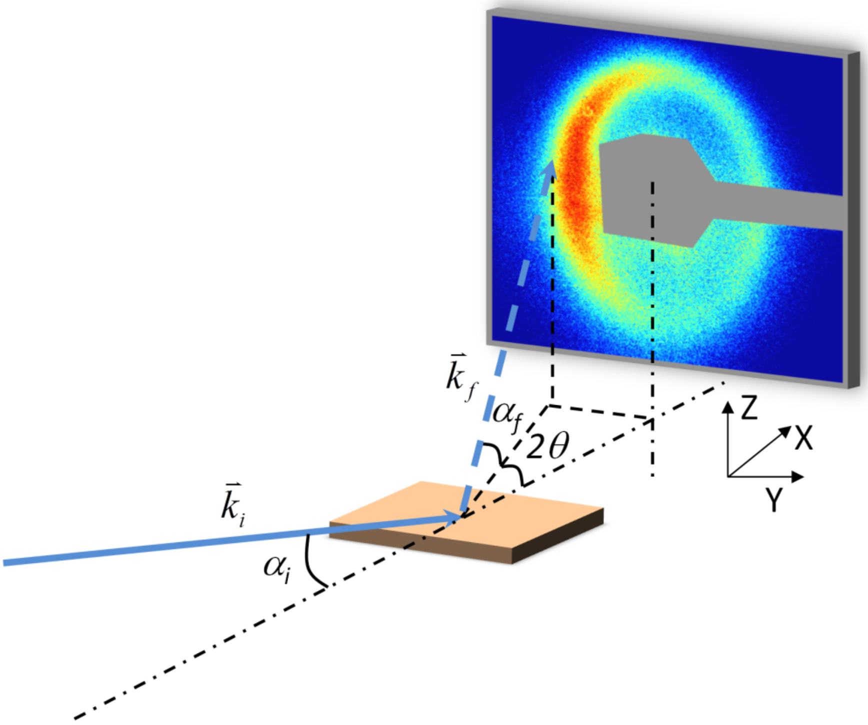

Supplementary Figure S1: *Schematics of the reflection scattering geometry. $\vec{k}_i$ denotes the wavevector of the incident X-ray beam and $\vec{k}_f$ the wavevector of the scattered beam deteceted under angles α_f and 2θ.*

The resonant magnetic scattering factor of a single Co atom for circular polarization of final and incident waves $\vec{\varepsilon}_{f,+}$, $\vec{\varepsilon}_{i,+}$ can be expressed as

$$f_n = \left(\vec{\varepsilon}^{*}_{f,+} \times \vec{\varepsilon}_{i,+}\right) \cdot \vec{m}\ \left(F^1_{+1} - F^1_{-1}\right) = \left[\frac{i}{2}\left(\hat{k}_f + \hat{k}_i\right) \cdot \vec{m} + \frac{1}{2}\left(\hat{k}_f \times \hat{k}_i\right) \cdot \vec{m}\right]\ \left(F^1_{+1} - F^1_{-1}\right), \tag{S1}$$

where $\vec{m}$ is the directon of magnetization, $\hat{k}_{i,f}$ the unit vectors of the wavevector transfers and F^L_M denotes the scattering amplitudes for resonant dipole transitions. The scattered intensity $I_{magn} = |f_n|^2$ for a single atom can be evaluated using (S1) with the unit wavevectors in the reflection geometry defined as following

$$\hat{k}_i = \begin{pmatrix} \cos(\alpha_i) \\ 0 \\ -\sin(\alpha_i) \end{pmatrix} \text{and} \quad \hat{k}_f = \begin{pmatrix} \cos(\alpha_f) \cdot \cos(2\theta) \\ \cos(\alpha_f) \cdot \sin(2\theta) \\ \sin(\alpha_f) \end{pmatrix}. \tag{S3}$$

The sample exhibits a perpendicular magnetic anisotropy with the magnetization vector pointing normal to the sample surface. In case the magnetization is directed along the z-axis of the scattering geometry the resulting scattering pattern is symmetric in 2θ.

A tilt of the direction of magnetization caused by a residual external magnetic field present at the sample position results in a non-symmetric scattering pattern (see Fig. S2) with respect to 2θ as observed in the experiment.

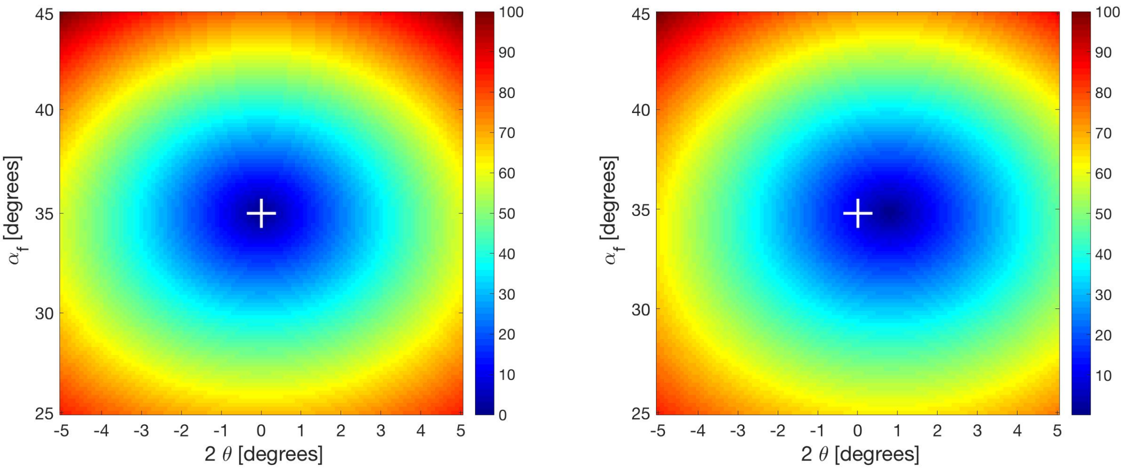

Supplementary Figure S2. *Plot of the magnetic form factor* I_{magn} *as a function of the two scattering angles* α_f *and* 2θ *for* α_i *= 35° and two different directions of magnetization with respect to the z-axis shown in Fig. S1. (a) The magnetization vector points along z-axis i.e.* $\vec{m} = (0{,}0{,}1)$ *or* $\vec{m} = (0{,}0,-1)$ *for up/ down domains . (b) shows the same calcualtion for* $\vec{m} = (0{,}0.1{,}1)$ *or* $\vec{m} = (0,-0.1,-1)$ *reflecting a titlting of the magnetization direction. The white cross indicates the origin of reciprocal space.*

2. Absorption of IR radiation

For a multilayer structure containing two alternating materials A and B with refractive indexes n_A and n_B, the profile for a single period can be described as $n = n_A + \alpha(n_B - n_A)$, where α is the concentration of material B in material A in the ith sublayer. The refractive index *n* is a complex value $\tilde{n} = n + ik$, where *n* and *k* are refractive index and extinction coefficient, respectively. For infrared light, the refractive indices can be well above one (for example, n = 2.5 for Co at 800 nm [1]). On the other side, in the X-ray region the refractive index is slightly less than one and usually expressed as $\tilde{n} = 1 - \delta + i\beta$.
For the calculation of the absorption of the IR pulse in the ML structure, we used the approach described in [2]. For the calculation of the electric field intensity $|E(z)|^2$ in the ML structure simulation we employed the Abeles matrix method [3] which is optimized for modelling of periodic multilayer structures using the amplitudes of the specular refracted E^+, and diffracted waves E^- as basis vectors for the calculation of reflectance and transmittance. A characteristic matrix describes the effects of the individual discontinuities (slice) and propagations of the entire discretized structure. The resulting matrix as a product of these characteristic matrices determines the distribution of the electromagnetic wave inside the multilayer system.
Next, we apply the equation $dA(z) = \alpha(z)n(z)|E(z)|^2dz$ to calculate the absorption profile inside the magnetic sample. The resulting functions for the IR radiation inside the magnetic multilayer structure are shown in Fig. S3.

[1] P. B. Johnson and R. W. Christy. *Optical constants of transition metals: Ti, V, Cr, Mn, Fe, Co, Ni, and Pd*, Phys. Rev. B 9, 5056-5070 (1974)
[2] A. R. Khorsand, M. Savoini, A. Kirilyuk & Th. Rasing, *Optical excitation of thin magnetic layers in multilayer structures* Nature Materials 13, 101–102 (2014)
[3] F. Abeles, "Sur la Propagation des Ondes Electromagnetiques dans les Milieux Stratifies," Ann. Phys. (Paris) 3, 504-520 (1948)

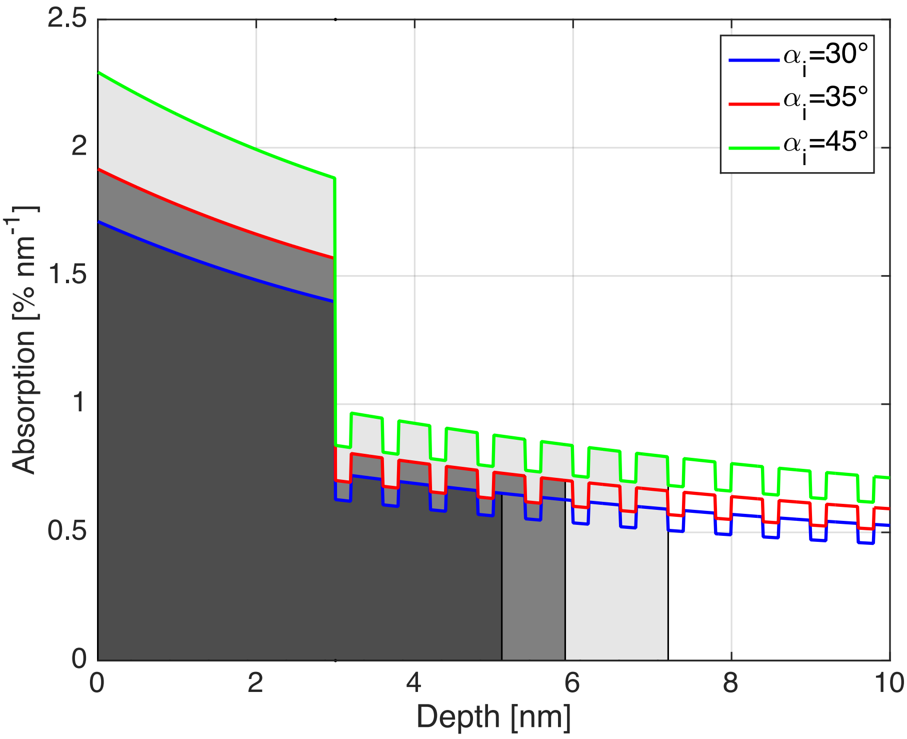

Supplementary Figure S3. *Absorption profile of the IR radiation as a function of depth inside the multilayer for the three different incident angles. The small ripples visible in the are caused by the Co/Pd layer structure. The Al capping layer extends to a depth of 3 nm. Vertical lines indicate the 1/e penetration depths of the XUV radiation. The grey areas represent the amount of IR power depsoited within the XUV penetration depths.*

α	of the scattering peak	penetration depth	depth in %
	Normalized to the value at α_i = 30°		
45°	1.28	1.65	10.6 %
35°	0.98	1.38	7.7 %
30°	1.00	1.00	6.4 %

Supplementary table ST1: *The table compares the maximum demagnetization (Figure 1(b)) and the IR power absorbed in the Co-Pd ML structure and the 3 nm Al cap layer at three incident angles α_i = 45°, 35°, 30°. The values are normalized to the value at α_i = 30°.*

3. Simulation of ultrafast spin transport through a magnetic domain network

The calculations of the time-dependent spatial arrangement of magnetizations for both spin channels are based on the following spin transport equation

$$\frac{\partial n^{\sigma}(E,\vec{r},t)}{\partial t}+\frac{n^{\sigma}(E,\vec{r},t)}{\tau^{\sigma}(E,t)}=\left(I-\frac{\partial}{\partial \vec{r}}\hat{\varphi}\right)S^{\sigma}_{eff}(E,\vec{r},t)$$

where $n^{\sigma}(E,\vec{r},t)$ is the distribution function for laser-excited electrons at time t, and spatial location $\vec{r}$ with energy E; $\tau^{\sigma}(E,t)$ is the lifetime, $\hat{\varphi}$ and I are the electron flux and identity operators, $S^{\sigma}_{eff}(E,\vec{r},t)$ is the effective source term for the hot electrons. The process of thermalization due to electron scatterings solves the time-dependent Boltzmann transport equation with a geometrically exact description.
For our simulation, we used two spatial coordinates: the *z* coordinate is normal to the layers, the *x* coordinate is defined as parallel to the layer. The spatial grid used is 0.002 nm.
In the present formulation it is assumed that the key parameters such as lifetimes and velocities of the electrons are spatially uniform inside each domain. At each time step, a solution of the differential equation is found for each domain separately. Using boundary conditions, we have calculated the evolution of the distribution function for the hot electrons inside the entire object.

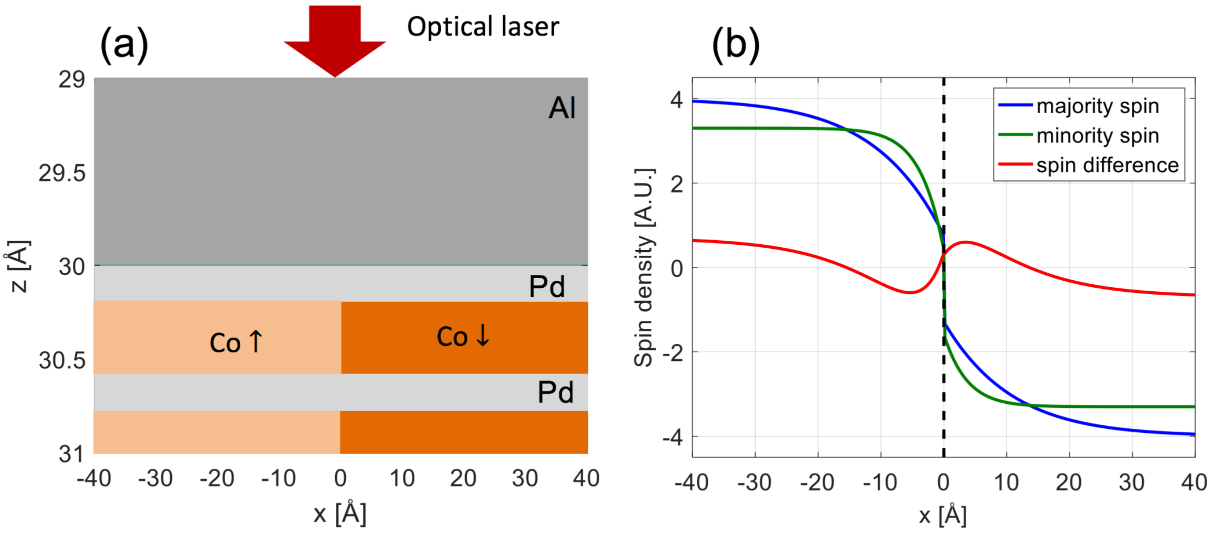

Supplementary Figure S4. *(a) Sample scheme for the ultrafast spin diffusion simulations. There is a 0.1 nm of Al cap layer and a spin up (Co↑) and spin down (Co↓) domains are considered with lateral width of 4 nm on both the sides of the domain boundary at x = 0. (b) Majority (blue) and minority (green) spin profiles and the spin difference profile (red) 40 fs after the arrival of the laser pump pulse. This is a typical example which shows that the spin difference can exhibit a magnetization-reversal near the domain boundary.*

The spin- and excitation energy-dependent electronic lifetimes and velocities, are taken from calculations in [1]. The laser-excited electron current from the Al cap layer is not spin polarized and has a random velocity direction; that is, it consists of equal amounts of spin-majority and minority electrons.

The spatial extensions of the magnetization-reversal are much less in our model than in the experimental data. In our model, we consider a uniform distribution of velocities and lifetimes within the domain for both majority and minority spins. We consider two neighboring domains and when an electron passes through an interface its velocity changes instantly from the majority to the minority or vice versa. Thus, the velocity function has the form of a step function. In reality, this function will have an error-function form.
The exact dimensions of this domain boundary region depend sensitive on the flux density and energy of the minority electrons and can easily stretch to tens of nanometers. In this region the velocities and lifetimes of the hot electrons also exhibit a spatially inhomogeneous distribution and depend on the electron concentration. Therefore, our model is only qualitatively in agreement with the data but a quantitative agreement would require a more complex simulation which is out of the scope of the present manuscript.

[1] Battiato, M. 2013. *Superdiffusive Spin Transport and Ultrafast Magnetization Dynamics: Femtosecond spin transport as the route to ultrafast spintronics*. Acta Universitatis Upsaliensis. Digital Comprehensive Summaries of Uppsala Dissertations from the Faculty of Science and Technology 1061. 62 pp. Uppsala. ISBN 978-91-554-8722-5.

4. Modelling of resonant magnetic scattering patterns

The structure factors for the unpumped (blue) and pumped (green) domain system is calculated as Fourier transform of the corresponding spin difference profiles via

$$S_0(q_r) = \left[\mathcal{F}\left((m_\uparrow(x) - m_\downarrow(x))_{unpumped}\right)\right]^2 \quad \text{(S4)}$$

$$and$$

$$S(q_r) = \left[\mathcal{F}((m_\uparrow(x) - m_\downarrow(x))_{pumped})\right]^2 \quad \text{(S5)}$$

where $\mathcal{F}()$ denotes the Fourier transform (see Fig S5a).
Using those expressions the distortion function $G(q_r)$ (red) is calculated as

$$G(q_r) = \frac{S(q_r)}{S_0(q_r)}. \quad \text{(S6)}$$

The shaded rectangle in Fig. S5(b) indicates the typical range of reciprocal space covered in the experiment. Knowing the average size of the domains of 190 nm from the position of the measured scattering peak in reciprocal space, the X-axis in (a) and q_r axis in (b) are scaled and are used for Figure 2.

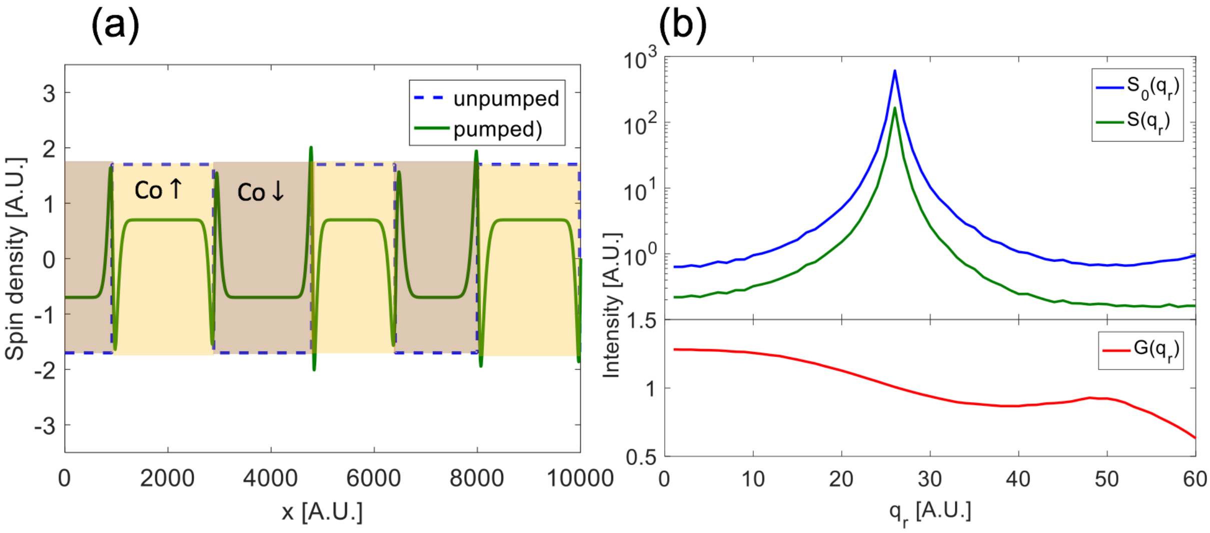

Supplementary Figure S5. *Modelling the distortion function $G(q_r)$. (a) An example of a spin difference profile $m_\uparrow(x) - m_\downarrow(x)$ over several Co↑ and Co↓ domains for an unpumped (blue) and a pumped (green) system (only a few domains are displayed). (b) The corresponding structure factors for the unpumped (blue) and pumped (green) systems. The resulting distortion function $G(q_r)$ is shown as red line.*

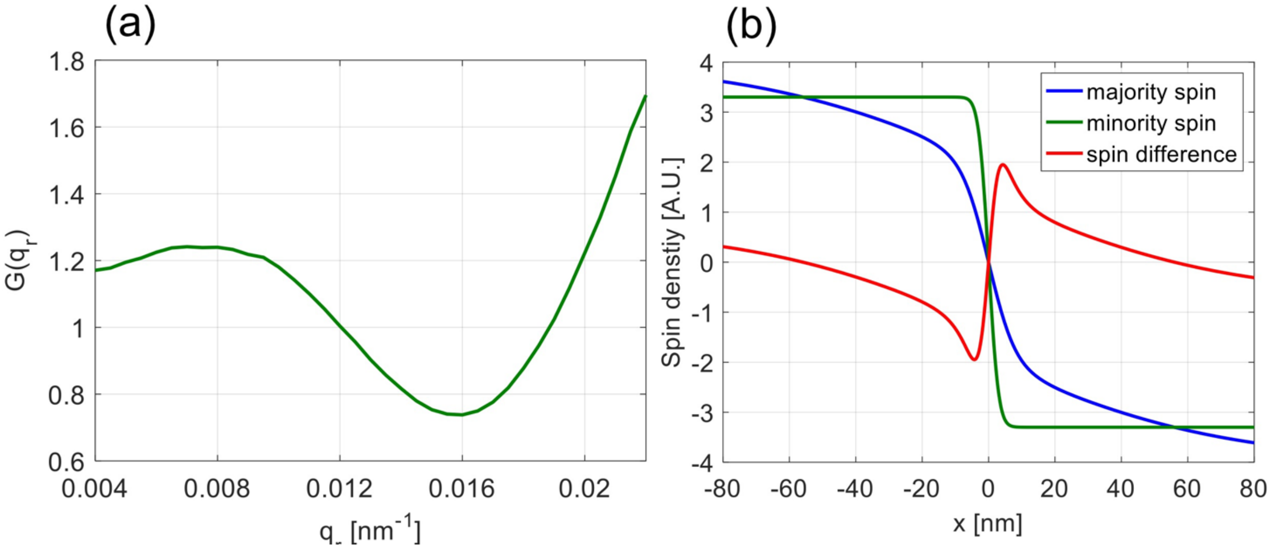

Supplementary Figure S6. *An example of a more pronounced modulation in $G(q_r)$. (Right) A spin profile consisting of a superposition of erf profiles leading to a more complex spatial profile of the magnetization (domain boundary is at x=0 nm). The corresponding distortion function (left) displays an additional modulation in the small q-region.*

5. Additional data for 2.8 nm penetration depth

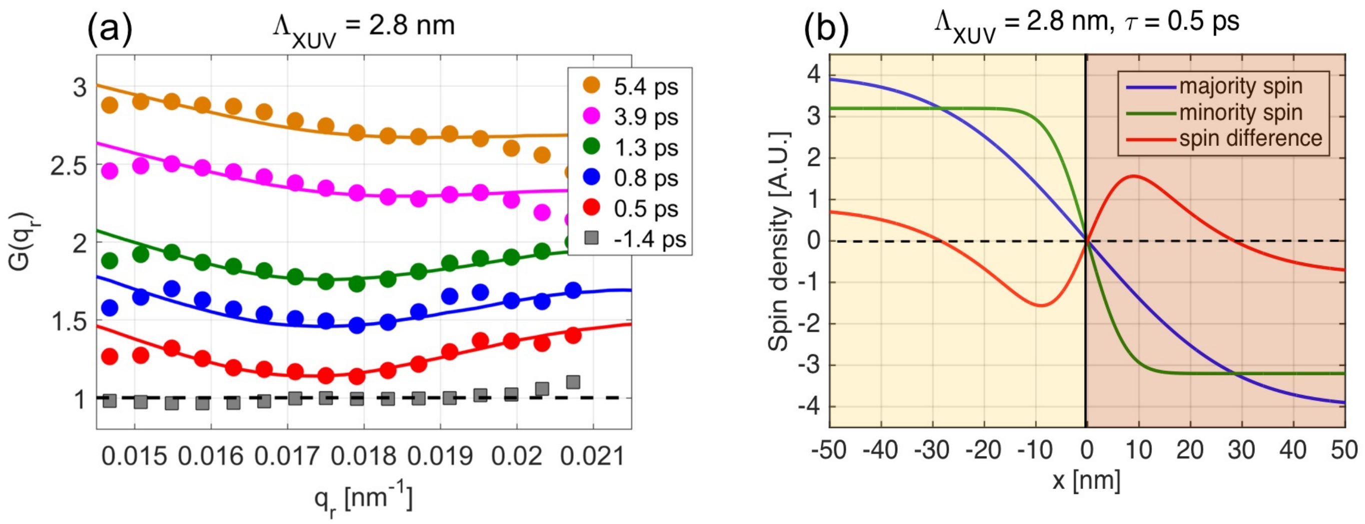

Supplementary Figure S7. *(a) The distortion functions from the measured structure factors (filled symbols) and the corresponding calculated distortion functions (solid lines) following the model described above for the XUV penetration depth Λ_{XUV} = 2.8 nm and at selected pump-probe delays. The distortion functions for different delays are vertically shifted for clarity. (b) The majority spin profile ($m_{\uparrow}(x)$), minority spin profile ($m_{\downarrow}(x)$) and the spin difference profile ($m_{\uparrow}(x) - m_{\downarrow}(x)$) at pump-probe delay of 0.5 ps. The spin up domain (Co$\uparrow$) exists for $x < 0$ and the spin down domain (Co$\downarrow$) for $x > 0$ with the domain boundary being at x = 0.*

6. Influence of the domain wall thickness

We model the influence of a finite domain wall width DW by the following spin profiles $m_\sigma(\tau, x) = \mu_\sigma \cdot erf\left[\frac{x}{(W_D/2 + W_\sigma(\tau))}\right]$ with W_D being identical for majority and minority carriers.

We have chosen five values of W_D=1, 6, 10, 14 and 20 nm. For each value of DW we refined the diffusion constants in such a way that our model distortion function fits the measured one as shown in Fig 2b. We find that the line-shape of the spin-profile is quite sensitive to the domain wall thickness, especially the amount of magnetization reversal tends to decrease with increasing domain wall thickness. However, the magnetization reversal is still the necessary ingredient to explain the shape of G(q).

The diffusion constants decrease with increasing W_D thickness, values of the majority diffusion constants are D=0.3, 0.28, 0.26, 0.25, 0.19 nm^2/fs for the W_D thicknesses. The diffusion constants of the minority carriers also tend to decrease however a lower limit is more difficult to find with increasing domain wall thickness.

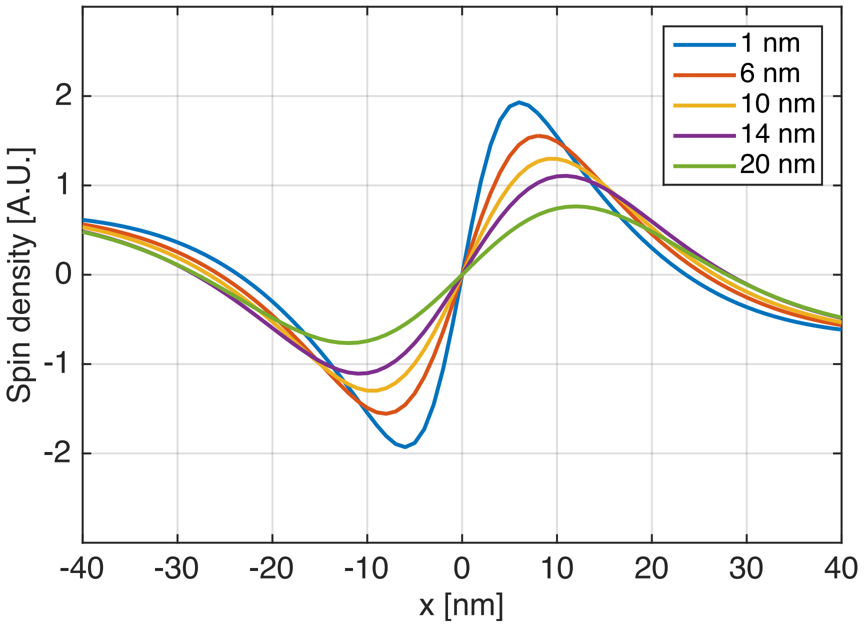

Figure S8. *Spin-density profiles yielding the same distortion function G(q) as shown in Fig. 2b for 0.5 ps delay but assuming different thickness of the domain walls varying from 1 nm to 20 nm.*

7. Spin current

We calculate the spin current with the help of the spin profiles of the majority carriers.
For a better statistic we first average the diffusion constants of all three XUV penetration depths by temporal binning. The resulting temperature dependence of the averaged diffusion constants is fitted by a phenomenological function. The result of this refinement is then used to calculate the transferred amount of spin density on a fine temporal grid. From this we finally calculate the spin current (Fig S9). Values below 0.5 ps are based on an extrapolation of our results towards smaller time scales.
The spin transfer peaks at around 0.5 ps and decreases noticeable within the first 1.5 ps when the electronic system reaches equilibrium with the lattice.

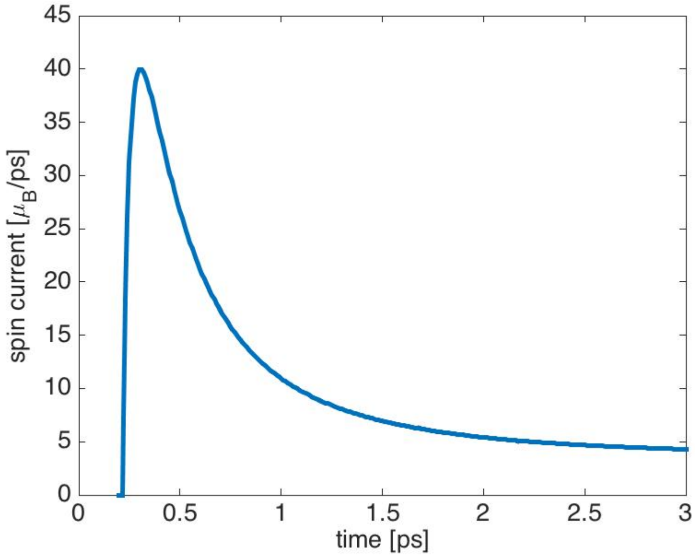

Figure S9. *Spin current of the majority carriers into the domain wall area as a function of time. The calculation is based on the averaged diffusion constants.*
